# Supershift properties for nonanalytic signals

**DOI:** 10.1515/nanoph-2024-0718

**Published:** 2025-04-30

**Authors:** Fabrizio Colombo, Irene Sabadini, Daniele Carlo Struppa, Alain Yger

**Affiliations:** Department of Mathematics, Politecnico di Milano, Milano, Italy; 293785 Chapman University Schmid College of Science and Technology, One University Drive, 92866, Orange, CA, USA; University of Bordeaux, Talence, Nouvelle-Aquitaine, France

**Keywords:** superoscillations, supershift, analyticity

## Abstract

The phenomenon of superoscillations is of great interest in microscopy, antenna design, and material sciences. This phenomenon has been generalized and has given rise to the concept of supershift, which is a far reaching extension that applies to functions that may present discontinuous derivatives. From this perspective, this is a notion that might have significant applications. This paper will provide an up to date report on the complex connections between the concept of supershift and that of analyticity.

## 1 Introduction

While the theory of superoscillations has been around for a long time, and it has already established both its impact for practical applications and its interesting role in the mathematics of infinite order and convolution operators, there are still many open problems that require attentive scrutiny. One of them is the complex relation between supershift (an important generalization of the concept of superoscillations) and analyticity. With the expression supershift one usually refers to functions that, if known on a countable number of points near the origin, are completely determined throughout the real line, or at least outside the sampling interval. This concept, in a way, seems to be a form of analyticity, if the countable number of points is collapsed to be the infinite derivatives in the origin. And yet, one can show that supershift can occur even in absence of analyticity, a feature that might have promising practical applications. Indeed, signals often lack analyticity, and the ability to predict their global behavior from local information (an essential component of superoscillatory functions) may play an interesting role in applications. This is, therefore, a question not only of great mathematical interest but also of potential practical impact when studying signals that do not have analytical properties and that exhibit corner or abrupt changes in their derivatives.

In this short report, we will provide a review and an update on the research that the authors have conducted (and continue to conduct) to elucidate this relationship. Detailed statements, proofs, and appropriate references can be found in [1], [2], [3].

### 1.1 Acknowledgment and dedication

The authors express their gratitude to the organizers of the conference for inviting them to present their results. They wish to dedicate this contribution in honor of Professor Capasso’s 75th birthday, and his fundamental contributions to science.

## 2 Superoscillating sequences: the basics

**Figure 1:**
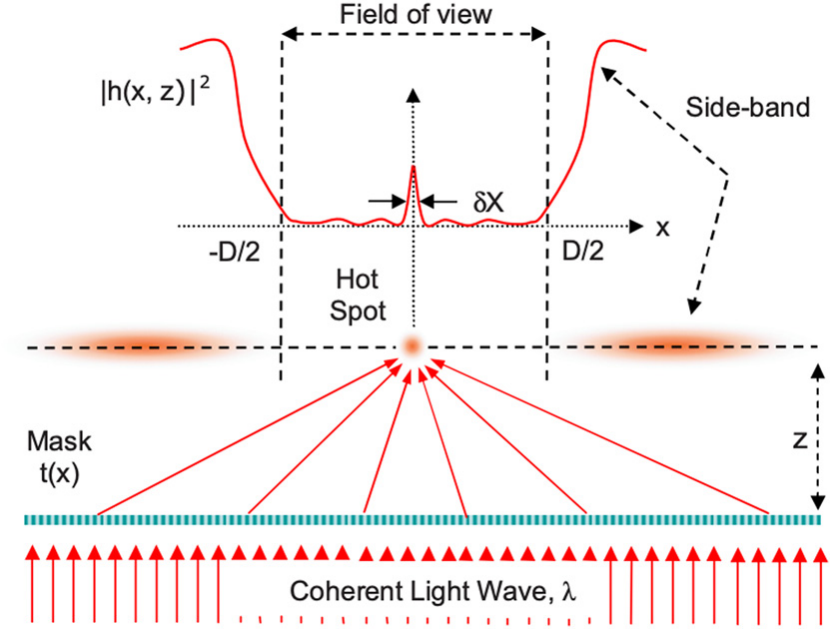
See Figure 1 in Huang and Zheludev [6]. (Copyright 2009 American Chemical Society)

The first definition of superoscillating functions goes back to [4] and can be given as follows: *superoscillating functions are bandlimited functions, which can oscillate faster than the highest frequency that they contain*, as we read in [5]. This definition requires a bit of context as, taken verbatim, seems almost a paradox. We will clarify this in the next few pages, but to begin with, we include a picture from [6] (Figure 1) that explains, in a very direct way, the importance of superoscillations for material sciences. As the reader can see, the coherent light of wavelength *λ* is cleverly superimposed, by using a screen with equally spaced microscopic holes, to generate what is known as a hot spot, in a very narrow region; this hot spot can be used, for example, to generate superresolution phenomena.

In order to clarify both how such superoscillations arise and the meaning of the intuitive definition we have given above, we consider the example from Aharonov’s original paper [4]. Let *x* be a real variable, *a* a real parameter, which we will take to be larger than 1. Consider the sequence of functions

$$F_{n}\left(x,a\right)≔{\left(\cos\frac{x}{n}+ia\sin\frac{x}{n}\right)}^{n},$$

which we easily see it is convergent to e^i*ax*^ and uniformly so on every compact set of the real line. An immediate calculation utilizing the Euler’s definition of the trigonometric functions and the binomial theorem also shows that *F*_*n*_(*x*, *a*) can be rewritten as

(2.1) $$\begin{array}{ll} F_{n}\left(x,a\right) & ={\left(\cos\left(\frac{x}{n}\right)+ia\sin\left(\frac{x}{n}\right)\right)}^{n}=\overset{n}{\underset{j=0}{\sum }}C_{j,n}\left(a\right){\text{e}}^{\text{i}\left(1-2j/n\right)x}, \end{array}$$

where $C_{j,n}\left(a\right)≔\left(\begin{array}{c} n\\ j \end{array}\right){\left(\frac{1+a}{2}\right)}^{n-j}{\left(\frac{1-a}{2}\right)}^{j}$ . This rewriting of the original functions is illuminating because we now see that we have a sequence of exponential sums, with frequencies $1-\frac{2j}{n}$ bounded between −1 and 1, that nevertheless converges to an exponential can be chosen with arbitrary large frequency *a*. The reason why this is possible is obviously because the coefficients in the sums *F*_*n*_ that appear in (2.1) depend on *n*, so that when we take the limit as *n* goes to infinity, we do not have a series, something that would violate the basic tenets of Fourier analysis.

A reasonable question, especially in view of applications, is whether this high energy waves can be produced only using these special exponentials with the very clearly specified frequencies $1-\frac{2j}{n}$ . The answer is negative. Indeed, in [7], we show that it is possible to create superoscillating functions with arbitrary frequencies *k*_*j*,*n*_, j=0,…,n;n∈N , as long as |*k*_*j*,*n*_| ≤ 1 and *k*_*i*,*n*_ ≠ *k*_*j*,*n*_ whenever *i* ≠ *j*. Specifically, if we choose

$$D_{j,n}\left(a\right)≔\overset{n}{\underset{t=0,t\neq j}{\prod }}\frac{k_{t,n}-a}{k_{t,n}-k_{j,n}}$$

then the sequence

$$f_{n}\left(x;a\right)≔\overset{n}{\underset{j=0}{\sum }}D_{j,n}\left(a\right)e^{ik_{j,n}x}$$

is superoscillating and converges to e^i*ax*^.

## 3 Toward supershift: basic definitions

The notion of supershift arises naturally in concrete situations (as we will see in a moment) and corresponds to a different way to interpret superoscillations. In the previous section, we have considered *f*_*a*_(*x*) ≔ e^i*ax*^ as a function of *x*, and where *a* is simply a parameter; we have then noticed that, if *a* is large, we can obtain such a highly oscillating function as limit of band-limited functions. One can, however, change perspective and consider the function *f*_*x*_(*a*) ≔ e^i*ax*^ as a function of *a*, with now *x* as a parameter. In this case, the notion of superoscillation shows us that we can find the value of *f*_*x*_(*a*) in an arbitrary point a∈R if we know its values in infinitely many points $\left\{\lambda_{j,n}=\left(1-\frac{2j}{n}\right)\right\}$ , or arbitrary {*k*_*j*,*n*_}, for *j* = 0, …, *n* and *n* = 1, …, + ∞. This is not anymore a question of frequencies or oscillations, but rather the interesting property that a function can be determined in a point far away from the origin by simply knowing its values near the origin.

A function satisfying the condition we have just described is said to display the *supershift* property. More formally we can say:

**Definition 1.**

(Supershift Property ${\left(SP\right)}_{F}$ ) Let *A* be an open interval in R , [−1, 1] ⊂ *A*, let ψ:A→F be a continuous map to a topological vector space F , and let

$$T_{H,n}\left[\lambda \right]\left(x\right)=\overset{n}{\underset{j=0}{\sum }}C_{H,j,n}\left(\lambda \right)e^{ixh_{j,n}}$$

be a superoscillating sequence. Then we say that *ψ* satisfies ${\left(SP\right)}_{F}$ on *A* with respect to $\left\{T_{H,n}\left[\lambda \right]:\;\lambda \in R\right\}$ if

$$\underset{n\to \infty }{\lim}\overset{n}{\underset{j=0}{\sum }}C_{H,j,n}\left(a\right)\psi \left(h_{j,n}\right)=\psi \left(a\right).$$

There are obviously several possible interesting choices for the space F ; among those, we will highlight the space F=E of infinitely differentiable functions, the space F=A of analytic functions, the space $F=D^{\prime }$ of distributions, and the space F=B of hyperfunctions. The choice of space to be used is generally speaking dictated by the problem itself, as we will show in the next example.

Here, we consider a very natural problem, which consists in evolving a superoscillating wave through the Schrödinger equation associated to the harmonic oscillator. The original wave displays the supershift phenomenon in the space of infinitely differentiable functions, for example, but as we will see once we evolve the wave, we lose the superoscillatory behavior and acquire a more subtle supershift in the space of distributions.

Consider then the prototypical superoscillating function *F*_*n*_(*x*, *a*) be as above. Take now *F*_*n*_(*x*, *a*) as initial value for the Cauchy problem for the Schrödinger equation for the harmonic oscillator, namely

$$i\frac{\partial \psi \left(x,t\right)}{\partial t}=\left[-\frac{\partial^{2}}{\partial x^{2}}+x^{2}\right]\psi \left(x,t\right),\;\;\;\;\psi \left(x,0\right)=F_{n}\left(x,a\right).$$

In [8], we show that the solution of this problem is given by

$$\begin{array}{ll} \psi_{n}\left(x,t\right) & =\overset{n}{\underset{j=0}{\sum }}\frac{C_{j,n}\left(a\right)}{\sqrt{\cos t}}\exp\left(-\frac{i}{2}\left(x^{2}+\lambda_{j,n}^{2}\right)\tan t+i\lambda_{j,n}\frac{x}{\cos t}\right), \end{array}$$

and that

$$\begin{array}{ll} \underset{n\to +\infty }{\lim}\psi_{n}\left(x,t\right) & =\frac{1}{\sqrt{\cos t}}\exp\left(-\frac{i}{2}\left(x^{2}+a^{2}\right)\tan t\right.\left.+ia\frac{x}{\cos t}\right). \end{array}$$

This indicates that the result of the evolution is a sequence that is not, strictly speaking, superoscillatory, but yet it exhibits the supershift property in the sense that if we parametrize it as a family of functions depending on the parameter *λ* as in

$$\psi \left[\lambda \right]\left(x,t\right)=\frac{1}{\sqrt{\cos t}}\exp\left(-\frac{i}{2}\left(x^{2}+\lambda^{2}\right)\tan t+ia\lambda \frac{x}{\cos t}\right)$$

we see that the knowledge of its values on a family of *λ*s near the origin is sufficient to determine its value for an arbitrary large value of *λ* = *a*. In addition, we can see clearly (because of the singularities that arise) that such a supershift property must be considered within the framework of hyperfunctions (distributions are not quite enough as the singularities of the holomorphic functions that extend the solutions are of essential and not polar nature).

We close this section with some definitions that refine those given before, and that will be necessary to understand the results of the next section.

**Definition 2.**

(*H*-trigonometric polynomials). Let $H={\left\{h_{j,n}\right\}}_{j=0,\ldots ,n;n=1,\ldots ,\infty }$ be a sequence of real numbers, and let $C_{H,j,n}\in C$ . Then, an *H*-sequence of trigonometric polynomials is a sum of the form

$$T_{H,n}\left(x\right)=\overset{n}{\underset{j=0}{\sum }}C_{H,j,n}e^{ixh_{j,n}}.$$

**Definition 3.**

((*A*, *H*)-sequence of trigonometric polynomials). Given an open subset *A* in R , an (*A*, *H*)-sequence of trigonometric polynomials *T*_*H*,*n*_[*a*] is an *A*-parametrized *H*-sequence of trigonometric polynomials with *C*_*H*,*j*,*n*_(*a*) a continuous function of *a* ∈ *A*.

**Definition 4.**

(Superoscillating sequence). An (*A*, *H*)-sequence of generalized trigonometric polynomials is *superoscillating* if

$$\underset{n\to \infty }{\lim}T_{H,n}\left[a\right]\left(x\right)=e^{i\mathrm{ax}}$$

**Example 5.**

The sequences

$$F_{n}\left(x,a\right)=\overset{n}{\underset{j=0}{\sum }}C_{j,n}\left(a\right){\text{e}}^{\text{i}\left(1-2j/n\right)x}$$

and

$$f_{n}\left(x,a\right)=\overset{n}{\underset{j=0}{\sum }}D_{j,n}\left(a\right)e^{ik_{j,n}x}$$

are both (R,H) -superoscillating with respect to *H* = {*λ*_*j*,*n*_ ≔ 1 − 2*j*/*n*} and to *H* = {*k*_*j*,*n*_}, respectively.

With these formal definitions, we can now say that if, for any a∈R , we call *ψ*[*a*] the C -valued distribution

$$\psi \left[a\right]\left(x,t\right)={\left(\cos t\right)}^{-\frac{1}{2}}\exp\left(-\frac{i}{2}\left(x^{2}+a^{2}\right)\tan t+ia\frac{x}{\cos t}\right),$$

then $\psi :R\to B^{\prime }$ enjoys ${\left(SP\right)}_{F}$ for $F=B\left(R\times R^{+},C\right)$ .

**Remark 1.**

The property of supershift says, roughly speaking, that if you know infinitely many values of a function *ψ* near the origin, then we can obtain the value of *ψ* in a larger region; such value can be explicitly calculated as a limit of values obtained at the various points. On the other hand, a function is analytic if when we know all of its derivatives in the origin (infinitely many values at the same point), then we can find the value of the function in a larger region; such value is calculated as limit of the values obtained through the infinitely many derivatives. It is, therefore, natural to ask whether there is any relationship between these two properties.

## 4 Analyticity implies supershift

This section will present a first approach to the question of the relationship between the two concepts and will demonstrate that in fact analyticity implies supershift.

**Definition 6**

(Regular Sampling). Let {*ϵ*_*n*_} be a sequence of numbers in [0,1] that converges to zero. A *regular sampling* is the sampling associated to the sequence

$$H_{j,n}^{\epsilon }≔\left\{1-2\left(\frac{j+\epsilon_{n}\left(n-j\right)}{n}\right)\right\}.$$

**Proposition 2.**

The sequence

$$T_{n}^{\epsilon }\left[a\right]\left(z\right)={\text{e}}^{\text{i}\epsilon_{n}z}{\left(\cos\left(z\frac{1-\epsilon_{n}}{n}\right)+ia\sin\left(z\frac{1-\epsilon_{n}}{n}\right)\right)}^{n}$$

is superoscillating with convergence in the space Exp(C) of entire functions of exponential type.

The key argument to show that analyticity implies supershift is to look back at (and reformulate in parametric terms, see [1]) a classical result of Serge Bernstein on Bernstein polynomials and their use in approximating holomorphic functions.

**Theorem 7**

(Bernstein’s theorem [9]). Let *c* ∈ [0, 1], *ρ* > max(*c*, 1 − *c*) and let $\left\{G_{i}:\;i\in N\right\}$ be a bounded family (in H(D(c,ρ)) ) of holomorphic function in the disk. Let $\left\{\epsilon_{\ell }=\left(\epsilon_{\ell ,n}\right):\;\ell \in N\right\}$ be a family of sequences that converge to zero uniformly with respect to *ℓ*. Then for any *i* and *ℓ*, the sequence of polynomial maps

$$\overset{n}{\underset{j=0}{\sum }}\left(\begin{array}{c} n\\ j \end{array}\right)z^{j}{\left(1-z\right)}^{n-j}G_{i}\left(j\frac{1-\epsilon_{\ell ,n}}{n}\right)$$

converges, in H(D(c,ρ)) to *G*_*i*_(*z*), and the convergence, on each compact set, is uniform with respect to *i* and *ℓ*.

**Theorem 8**

(Analyticity implies supershift). Let *F* be an entire function on C . Then its restriction $\psi =F_{|R}$ to R satisfies a strong version of the supershift property with respect to the superoscillating sequence $\left\{T_{j}^{\epsilon }\left[a\right]\right\}$ for any sequence *ϵ* converging to zero.

The previous result (and its proof) shows that analyticity implies a strong version of supershift, which we have called *regular supershift*. It implies that, in addition to satisfying the usual supershift property, it behaves well with respect to translation so that

$$\begin{array}{ll} & \overset{n}{\underset{j=o}{\sum }}\left(\begin{array}{c} n\\ j \end{array}\right){\left(\frac{1+a}{2}\right)}^{n-j}{\left(\frac{1-a}{2}\right)}^{j}\psi \\ & \;\times \left(a^{\prime }+\left(1-2\left(\frac{j+\epsilon_{\ell ,n}\left(n-j\right)}{n}\right)\right)\right) \end{array}$$

converges uniformly to *ψ*(*a* + *a*′).

The important consequence of the result is that every real analytic function, provided it can be extended as a holomorphic function to a sufficiently large complex domain containing *A*, provides an example of regular supershift in *A*. In case *A* is unbounded, this is true for all restrictions of entire functions. Does the converse hold? In other words, is it true that every supershift inherits anayticity?

## 5 Supershift does not imply analyticity

We prove in this section that the general answer to the question highlighted at the end of the previous section is negative, and that the notion of supershift needs to be strengthened if one expects it to yield analyticity. This result, from the perspective of possible applications, is a positive one, as it shows that we can have the supershift phenomenon even for functions that admit singular derivatives.

**Theorem 9**

(Supershift does not imply analyticity). Let *G* ^±^ be two entire functions such that

$$G^{-}\left(0\right)=G^{+}\left(0\right),\;\frac{dG^{-}}{dz}\left(0\right)\neq \frac{dg^{+}}{dz}\left(0\right)$$

and define g:R→C as *g*(*b*) = *G* ^−^(*b*) if *b* ≤ 0 and *g*(*b*) = *G* ^+^(*b*) if *b* ≥ 0. Then there is an open interval A⊂R , with [−1, 1] ⊂ *A* such that the map *ψ*(*a*) = *g*(*a*) is a regular supershift.

In view of the previous result, even regular supershift does not imply analyticity. However, one may hope to prove a weaker statement, namely that the supershift property near one of the extremities of the interval [−1, 1] forces local analyticity in a convenient neighborhood of this extremity point.

**Remark 3.**

Everything we have seen up to now is based on the notion of *regular* sampling. So, for example, we have that restrictions of entire functions enjoy regular supershift properties. But what if we don’t use regularity on the sampling? We will, therefore, consider now the case in which *H* = {*h*_*j*,*n*_} is arbitrary, still within the constraint that |*h*_*j*,*n*_| ≤ 1.

**Proposition 4**

(Analyticity implies supershift). The sequence

$$T_{H,n}^{\mathrm{Lag}}\left[a\right]\left(z\right)≔\overset{n}{\underset{j=0}{\sum }}D_{j,n}\left(a\right)e^{ih_{j,n}z}$$

is superoscillating, see [7], with convergence in the space Exp(C) of entire functions of exponential type.

**Corollary 10.**

Any restriction to the real axis of an entire function enjoys regular supershift properties with respect to any (R,H) superoscillating sequence as in the previous proposition.

The main theorem given before relies on a beautiful idea of Kantorovich, for which we refer the reader to the original article [10], as well as to Lorentz’s [11]. In order for us to be able to use his idea, we had to consider a parametric version that allowed us to modify it and show that it is possible to extrapolate nonreal analytic functions, from irregularly sampled values on [−1, 1] through a sequence of real-analytic functions.

As we noted above, these results are developed in the context of regular sampling, but in our [2], we demonstrated that the phenomenon of supershift (through irregular sampling) does not imply analyticity either.

This has led us to formulating a conjecture, to which next section is devoted.

## 6 A conjecture, and a partial answer

When looking at [1], [2], [3] as quickly summarized in the previous sections, we are led to formulating the following conjecture.

### 6.1 Conjecture

A continuous function *ψ* on the real line is a regular supershift if and only if it is the restriction to the real line of an entire function.

Two partial results seem to lead some credence to this conjecture (which is the object of our new studies).

**Theorem 11.**

Any restriction to the real axis of an entire function enjoys regular supershift properties with respect to any (R,H) superoscillating sequence as in the previous proposition.

More important, we have a partial converse, for which we offer here a sketch of a proof (the complete proof is in [3]).

**Theorem 12.**

Let *ψ* be a 2*π*-periodic continuous regular supershift on R . Then there is a 2*π*-periodic entire function on C of which *ψ* is the restriction to the real line.

**Proof.**

We offer here a sketch of the proof of this result in five steps. The first step consists in using the periodicity of *ψ* to write

$$\psi \left(a\right)=\underset{k\in Z}{\sum }\gamma_{k}e^{i\mathrm{ka}}.$$

In the second step, we consider the auxiliary function

$$\varphi \left(a\right)≔\frac{1}{2\pi }\overset{2\pi }{\underset{0}{\int }}\psi \left(a-\tau \right)\psi \left(\tau \right)d\tau =\underset{k\in Z}{\sum }\gamma_{k}^{2}e^{i\mathrm{ka}}.$$

and we show that it is still a 2*π*-periodic continuous regular supershift on R . Step three consists in showing that, for any *R* > 1, and any *a*′ ∈ [0, 2*π*],

$$\varphi \left(a^{\prime }+R\right)=\underset{n\to \infty }{\lim}\underset{k\in Z}{\sum }\gamma_{k}^{2}{\left(\cos\left(k/n\right)+iR\sin\left(k/n\right)\right)}^{n}e^{ika^{\prime }}.$$

One now use (step four) Parseval’s formula to show the existence of a constant *C*_*φ*_(*R*) such that for *w* = *k*/*n*

$$\sum |\gamma_{k}{|}^{4}{\left|\cos w+iR\sin w\right|}^{2n}\leq C_{\varphi }\left(R\right).$$

To conclude the proof we use suitable trigonometric estimates (see [7]) to finally obtain that for any *R* > 0 we have

$$\sum |\gamma_{k}|e^{R|k|},$$

which implies that *ψ* extends to an entire 2*π*-periodic function on C .□

## 7 Some final remarks

We conclude this short survey with a few remarks. The notion of superoscillations arises naturally in the context of quantum weak values; when evolving superoscillating waves, however, a different phenomenon emerges, that we have called supershift, and that resembles, in spirit, the notion of analyticity.

While it is not difficult to show that analyticity implies supershift, it is more delicate to demonstrate that not only analyticity is a stronger property than supershift but also that the exact nature of the relationship between analyticity and supershift is more complex than one could expect to begin with.

From a mathematical point of view, the instruments that proved helpful in understanding this relationship are essentially interpolation arguments, whether using Lagrange interpolation or Bernstein polynomials. Some very interesting new phenomena appear when these ideas are extended to the case of several variables.

One can foresee applications of these ideas to the study of nonanalytic signals, to which we can apply the notion of supershift.
